# Recombinant Monovalent Llama-Derived Antibody Fragments (VHH) to Rotavirus VP6 Protect Neonatal Gnotobiotic Piglets against Human Rotavirus-Induced Diarrhea

**DOI:** 10.1371/journal.ppat.1003334

**Published:** 2013-05-02

**Authors:** Celina G. Vega, Marina Bok, Anastasia N. Vlasova, Kuldeep S. Chattha, Silvia Gómez-Sebastián, Carmen Nuñez, Carmen Alvarado, Rodrigo Lasa, José M. Escribano, Lorena L. Garaicoechea, Fernando Fernandez, Karin Bok, Andrés Wigdorovitz, Linda J. Saif, Viviana Parreño

**Affiliations:** 1 Instituto de Virología, Centro de Investigaciones en Ciencias Veterinarias y Agronómicas, INTA Castelar, Buenos Aires, Argentina; 2 Food Animal Health Research Program, The Ohio Agricultural Research and Development Center, Veterinary Preventive Medicine Department, The Ohio State University, Wooster, Ohio, United States of America; 3 Alternative Gene Expression S.L. (ALGENEX), Centro Empresarial, Parque Científico y Tecnológico de la Universidad Politécnica de Madrid, Campus de Montegancedo, Pozuelo de Alarcón, Madrid, Spain; 4 Departamento de Biotecnología. Instituto Nacional de Investigación y Tecnología Agraria y Alimentaria (INIA), Madrid, Spain; 5 Caliciviruses Section, Laboratory of Infectious Diseases, National Institute of Allergy and Infectious Diseases (NIAID), National Institutes of Health (NIH), Bethesda, Maryland, United States of America; North Carolina State University, United States of America

## Abstract

Group A Rotavirus (RVA) is the leading cause of severe diarrhea in children. The aims of the present study were to determine the neutralizing activity of VP6-specific llama-derived single domain nanoantibodies (VHH nanoAbs) against different RVA strains *in vitro* and to evaluate the ability of G6P[1] VP6-specific llama-derived single domain nanoantibodies (VHH) to protect against human rotavirus in gnotobiotic (Gn) piglets experimentally inoculated with virulent Wa G1P[8] rotavirus. Supplementation of the daily milk diet with 3B2 VHH clone produced using a baculovirus vector expression system (final ELISA antibody -Ab- titer of 4096; virus neutralization -VN- titer of 256) for 9 days conferred full protection against rotavirus associated diarrhea and significantly reduced virus shedding. The administration of comparable levels of porcine IgG Abs only protected 4 out of 6 of the animals from human RVA diarrhea but significantly reduced virus shedding. In contrast, G6P[1]-VP6 rotavirus-specific IgY Abs purified from eggs of hyperimmunized hens failed to protect piglets against human RVA-induced diarrhea or virus shedding when administering similar quantities of Abs. The oral administration of VHH nanoAb neither interfered with the host's isotype profiles of the Ab secreting cell responses to rotavirus, nor induced detectable host Ab responses to the treatment in serum or intestinal contents. This study shows that the oral administration of rotavirus VP6-VHH nanoAb is a broadly reactive and effective treatment against rotavirus-induced diarrhea in neonatal pigs. Our findings highlight the potential value of a broad neutralizing VP6-specific VHH nanoAb as a treatment that can complement or be used as an alternative to the current strain-specific RVA vaccines. Nanobodies could also be scaled-up to develop pediatric medication or functional food like infant milk formulas that might help treat RVA diarrhea.

## Author Summary

Group A rotavirus (RVA) is the most common cause of severe diarrhea in human infants worldwide. Live-attenuated rotavirus vaccines are available to prevent rotavirus diarrhea in children, although their efficacy in impoverished areas has been questioned, in addition to not being suitable for children suffering from immune deficiencies. Since no rotavirus-specific treatments are available as an alternative, we investigated llama-derived single-chain antibody fragments (VHH) as preventive therapy and a potential treatment option. Gnotobiotic piglets were chosen as an animal model because their gastrointestinal physiology and mucosal immune system resemble that of human infants. We evaluated the broad neutralizing activity of a VHH clone (3B2) to different genotypes of RVA circulating in humans, and tested the efficacy of oral administration of 3B2 VHH as a functional milk to prevent the diarrhea induced by one of the most prevalent human RVA strains (G1P[8]). Supplementation of the milk diet with 3B2 twice a day for 9 days conferred full protection against rotavirus-associated diarrhea and significantly reduced virus shedding in gnotobiotic piglets experimentally inoculated with a human RVA. This study demonstrates the potential application of VHH to prevent rotavirus-induced diarrhea, and suggests that VHHs should be further investigated as a suitable treatment for gastroenteritis.

## Introduction

Diarrhea is the second most common cause of childhood mortality worldwide, causing 1.3 million deaths among children younger than 5 years of age [1]. Group A rotavirus (RVA) is the leading cause of severe diarrhea in children worldwide and is responsible for approximately 29% of all diarrheal deaths, causing 453,000 deaths per year [2]–[5]. Human rotaviruses (Group A, B and C) have also been implicated as causative agents of diarrheal outbreaks occurring in nursing homes [6], among travelers [7], in day-care centers [8], and in patients suffering from a variety of immunodeficiency conditions [9], [10].

Rotaviruses have a genome consisting of 11 segments of double-stranded RNA. Most segments encode a single polypeptide, allowing the virus to express six structural proteins (VPs) and five non-structural proteins (NSPs) [11]. Twenty-seven G-types and 35 P-types that independently elicit virus-neutralizing antibodies (Abs) have been identified based on the RVA outer capsid proteins VP7 (G-type) and VP4 (P-type) [12]. Of these, G-types G1 to G4 and G9 combined with P-types P[4], P[6] and P[8] account for most of the G-P combinations of human RVA detected globally. They are responsible for approximately 90% of all RVA infections worldwide, showing different relative proportions by year and region [13]–[15]. Other genotypes such as G5, G8, G10 and G12 in combination with P[7], P[8] and P[9] were recently reported infecting children from South America, the Caribbean, Africa and Asia with lower incidences [15]–[21].

Currently, diarrhea caused by RVA can be prevented through vaccination but treatment strategies are non-specific, largely symptom-based, and involve fluid, electrolyte replacement and maintenance of nutrition [22]. Live-attenuated oral RVA vaccines have variable degrees of efficacy and a high cost [23], [24]. Recent clinical trials showed that RVA vaccines have significantly lower efficacy in countries with limited infrastructure and resources, usually the countries with the highest RVA burdens [24]. Moreover, vaccine-acquired human RVA infection and diarrhea has been previously reported in children suffering from severe combined immunodeficiency, which led the Centers for Disease Control to issue a recommendation against their use in this population [25]. On the other hand, rotavirus-specific treatments such as passive immunotherapy using antibodies (Abs) are associated with various degrees of success against infectious diseases of different etiologies, such as viral, bacterial, fungal and protozoal origin in both humans and animals [10], [22], [26]–[35]. Therefore, we investigated llama-derived single-chain antibody fragments (VHH) against rotavirus VP6 protein as preventive therapy and a potential treatment option. VHHs are the smallest molecules with antigen-binding capacity and present distinctive properties that differentiate them from conventional antibodies, such as the ability to remain intact in the gastrointestinal tract during oral administration.

The RVA VP6 inner capsid protein is highly immunogenic and constitutes the target antigen of most immunodiagnostic tests [36], [37]. Based on its nucleotide (nt) variability (85% nt identity among VP6 of the same group), at least 17 VP6 I (for intermediate capsid shell) genotypes have been described, I1 and I2 being the most frequently found in human RVA strains [38], [39]. Most studies show that conventional Abs to VP6 lack extracellular neutralizing activity *in vitro* [40]–[43]. Tosser *et al.* developed a monoclonal Ab to VP6 (RV-133) that interacted with both double- and triple-layer particles and induced partial decapsidation but failed to neutralize RVA *in vitro* [40]. Burns *et al.* reported two IgA monoclonal Abs to VP6 (clones 7D9 and 10C10) that did not present detectable neutralizing activity *in vitro* but inhibited viral transcription and completely protected against infection *in vivo* [44], [45]. However, conflicting results regarding the effectiveness of VP6 Abs mediating passive protection in neonatal mice have been reported [44], [46]. Some studies showed that VP6-specific maternal Abs did not provide passive protection against RVA-associated diarrhea in neonatal mice [47], [48] while others reported partial protection in neonatal mice born to dams immunized with recombinant VP6 derived from C486 RVA (G6P[1]I?) that did not develop virus neutralizing Abs [41]. Similar studies that used passive IgA monoclonal Abs to VP6 suggested that protection *in vivo* is in fact mediated by the binding of VP6 IgA Ab to RVA, which protects by intracellular neutralization during transcytosis in the mouse gut [44], [48]–[50]. In any case, it is well known that the continuous presence of high titers of passive RVA Abs in the gut lumen (naturally produced or artificially added to the milk) fully protects against diarrhea and significantly reduces virus shedding [43], [51]–[53]. We have previously developed VHH against VP6 from bovine RVA strain C486 (G6P[1]I?). These VHH nanoAbs neutralized RVA *in vitro*, independently of the serotype. This result was further confirmed *in vivo* by partial protection of VHH clones 3B2 and 2KD1 against RVA challenge in a neonatal mouse model, demonstrating for the first time the broad neutralization activity of VP6 specific VHH nanoAbs *in vitro* and *in vivo* [54], [55].

Neutralizing VHH nanoAbs directed to VP6 may provide a new strategy for the prevention and treatment of RVA diarrhea. However, being a heterologous source of Abs, the host immune response to the passive treatment must be considered, especially in view of plans to use llama VHH nanoAbs prophylactically and/or therapeutically in humans. We chose for this study the Improved Baculovirus Expression System (IBES technology), which uses baculovirus expression vectors in combination with *Trichoplusia ni* larvae as living biofactories. The benefits of this platform have been largely proven for other recombinant proteins as a cost-efficient production platform for a number of recombinant proteins [55]–[58]. The optimal production of high yields of fully functional VP6-specific VHH derived from larvae has been published recently, wherein the protective effect of this VHH nanoAb in mice was reported [55], confirming previous results obtained with VHH derived from *Escherichia coli* [54]. To test the VP6-specific VHH antibody against human RVA gastroenteritis we chose the neonatal gnotobiotic (Gn) pig model. The Gn piglets are a widely known animal model susceptible to human RVA infection and disease and the pathogenicity of RVA in Gn piglets mimics that after natural RVA infection of infants. Furthermore their gastrointestinal physiology and mucosal immune system resemble that of human infants [59]–[61].

Here, we assess the broad neutralizing activity of 3B2 VHH to different genotypes of RVA circulating in humans; and evaluate the passive protection conferred by oral administration of VP6-specific VHH clone 3B2 against RVA-induced diarrhea in neonatal Gn piglets experimentally inoculated with one of the most prevalent strains of human RVA, Wa G1P[8]I1.

## Results

### VP6-specific VHH nanoAbs protect against human RVA diarrhea and reduce virus shedding

The aim of the present experiment was to evaluate the protection of orally administered G6P[1] VP6-specific VHH against human RVA associated diarrhea in Gn piglets experimentally inoculated with virulent Wa G1P[8]I1 RVA, one of the most prevalent RVA strains circulating in human infants worldwide [15]–[21]. The experimental design included five groups of Gn pigs receiving milk supplemented with one of the following: 3B2 VHH nanoAbs (VHH produced against G6P[1]), Wa G1P[8]I1 human RVA-specific porcine IgG Abs (IgG), G6P[1] VP6-specific chicken IgY Abs (IgY), control larvae (CL) or Ab-free milk (No Treatment, NT). The results for VN and ELISA isotype-specific Ab titers to Wa human RVA in the supplemented milk are depicted in Table 1. As shown, VP6-specific VHH nanoAbs had a final Wa human RVA ELISA Ab titer of 4096 and a VN titer of 256 in the supplemented milk, similar to the Ab titers obtained in porcine anti-Wa human RVA IgG supplemented milk used as a positive homologous control treatment. Milk containing chicken IgY polyclonal Abs purified from eggs of VP6-vaccinated hens with a final Wa human RVA ELISA Ab titer of 4096 and a VN titer to Wa human RVA of 64 was considered as a control for heterologous conventional Abs to VP6. As expected, no Abs against Wa human RVA were detected for CL supplemented milk and Ab free milk -NT, consisting of sterile commercial bovine milk- (Table 1).

**Table 1:** VN and ELISA isotype-specific Ab titers to Wa HRV in the supplemented bovine milk used to feed Gn piglets.

Group name	Treatment group	Ab titer to HRV Wa P[8]G1	
		ELISA	VN a
**VHH**	**anti-VP6 VHH**	**4096**	256
		(VHH)	
**IgY**	**anti-VP6 IgY**	**4096**	64
		(IgY)	
**CL**	**control larvae**	**<4**	<4
		(neg)	(neg)
**IgG**	**anti-Wa HRV IgG**	**4096**	256
		(IgG)	
**NT**	**Ab free milk**	**<4**	**<4**
		(neg)	(neg)

a Determined by fluorescence focus forming unit reduction assay. A 10 ml (4.8 percent) aliquot of each Ab pool (VHH/IgY/Control larvae/IgG) was added to 210 ml of sterile commercial bovine milk to obtain the indicated final Ab titer to Wa HRV. Neg = negative.

Table 2 shows the presence of diarrhea and the amount of virus shedding detected. Supplementation of the milk diet with VP6-specific VHH nanoAbs for 9 days conferred full protection (5 of 5 animals protected) against Wa Human RVA diarrhea in Gn pigs. The positive control group, pigs treated with the same ELISA and VN titer of homologous IgG Abs to Wa human RVA, were partially protected, as 4 out of 6 animals did not develop diarrhea after virus inoculation. In the 2 piglets from this group that developed diarrhea, there was a delay in the onset (post inoculation day -PID-4; p = 0.0214) and a reduction in the mean duration (0.7 days; p<0.0001) and in the mean cumulative diarrhea score (4.0; p<0.0001), all of which differed significantly compared to animals that did not receive any treatment (NT group; no animals protected; mean onset: PID 2.2; mean duration: 6.8 days; mean cumulative diarrhea score: 15.6). Finally, animals in the IgY and CL groups developed diarrhea with statistically comparable protection parameters (in terms of percentage of animals with diarrhea, mean onset, duration and mean cumulative diarrhea score) to that of the NT group of pigs (Table 2).

**Table 2:** Diarrhea and virus shedding in Gn piglets after oral inoculation with VirHRV Wa (P[8]G1).

Treatment Group	n	Diarrhea^b^^,^ e				Virus shedding^a^^,^ e					
		N° Protected Animals^d^	Mean Onset (PID)	Mean Duration (days)	Mean Cumulative Score^c^	% Infected Animals	Mean Onset (PID)	Mean Duration (days)	AUC (FFU/ml *day)	Mean Peak Titer (log FFU)	Mean Shed Virus (log FFU)
**VHH**	5	5/5 ^A^	ND ^A^	ND ^A^	ND ^A^	5/5	2.6	3.0 ^AB^	6.1×10^4 AB^	4.2 ^AB^	0.8 ^AB^
**IgY**	4	0/4 ^B^	1.8 ^B^	4.0 ^BC^	8.3 ^BC^	4/4	1.8	5.3 ^BC^	2.3×10^5 BC^	5.2 ^AB^	1.3 ^B^
**CL**	4	0/4 ^B^	2.0 ^B^	4.5 ^C^	9.3 ^C^	4/4	1.8	4.0 ^ABC^	2.1×10^5 BC^	4.9 ^AB^	1.0 ^AB^
**IgG**	6	4/6 ^AB^	4.0 ^C^	0.7 ^AB^	4.0 ^B^	5/6	3.0	1.6 ^A^	1.9×10^4 A^	3.4 ^A^	0.5 ^A^
**NT**	5	0/5 ^B^	2.2 ^B^	6.8 ^C^	15.6 ^C^	5/5	2.2	5.6 ^C^	1.8×10^6 C^	5.7 ^B^	1.2 ^B^
**p-value**	-	<0.05	0.0214	<0.0001	<0.0001	-	-	0.0012	0.0055	0.014	0.0012

Treatment Group abbreviations as well as passive treatments administered to each group of pigs are clarified in Table 1. VHH = piglets treated with VHH nanoAbs to VP6 at a final Wa HRV ELISA Ab titer of 4096; IgY = piglets treated with IgY Abs to VP6 at a final Wa HRV ELISA Ab titer of 4096; CL = piglets treated with control larvae; IgG = piglets treated with porcine IgG Abs to Wa HRV at a final Wa HRV ELISA Ab titer of 4096; NT = not treated piglets; n = number of animals per group; AUC = area under the curve. ND = not detected. PID = post inoculation day.

a Determined by ELISA and CCIF.

b Diarrhea duration was defined as the number of days with fecal score ≥2. Stool consistency was scored daily (0 = normal; 1 = pasty; 2 = semi-liquid; 3 = liquid).

c Mean cumulative score was Σ(faecal scores ≥2)/number of animals. For statistical analysis, ND was considered as zero.

d Proportions in the same column with different superscript upper case letters (A, B, C) differs significantly (Fisher's exact test, p<0.05).

e Means in the same column with different superscript upper case letters differ significantly (Kruskall-Wallis rank sum test; p<0.05). Piglets were fed with 210 ml of sterile milk, twice a day, for 9 days with or without the addition of the corresponding volume of VP6 VHH pool, VP6 IgY pool, control larvae pool or Wa HRV porcine IgG pool, according to the experimental group.

Virus shedding was detected in all animals in the VHH group (5 out of 5 infected animals). Although the mean onset of virus shedding was similar in all groups of pigs, in the VHH group, the mean duration (3.0 days; p = 0.0012) and the AUC (6.1×10^4^ FFU/ml*day; p = 0.0055) were significantly lower than the ones in the NT group (mean duration: 5.6 days, AUC: 1.8×10^6^ FFU/ml*day). Mean peak titer and mean titer of virus shed showed a trend toward lower values than in the NT group, but not significantly so. For the IgG group, 5 of the 6 animals had only a short duration and low amounts of virus shedding (mean onset: PID 3.0; mean duration: 1.6 days; AUC: 1.9×10^4^ FFU/ml*day). The mean peak titer (10^3.4^ FFU/ml; p = 0.014) and mean titer of virus shed (10^0.5^ FFU/ml; p = 0.0012) for this group of pigs were significantly lower than pigs in NT group (10^5.7^ FFU/ml and 10^1.2^ FFU7ml respectively). The IgY and CL groups were intermediate between the positive control group (IgG group) and the NT pigs regarding mean duration, AUC, mean peak titer and mean titer of virus shed. There were no statistically significant differences in the onset of virus shedding (IgY = CL = PID 1.8) and the percentage of animals infected (IgY = CL = all the animals infected) compared to the NT group (Table 2).

**Figure 1:**
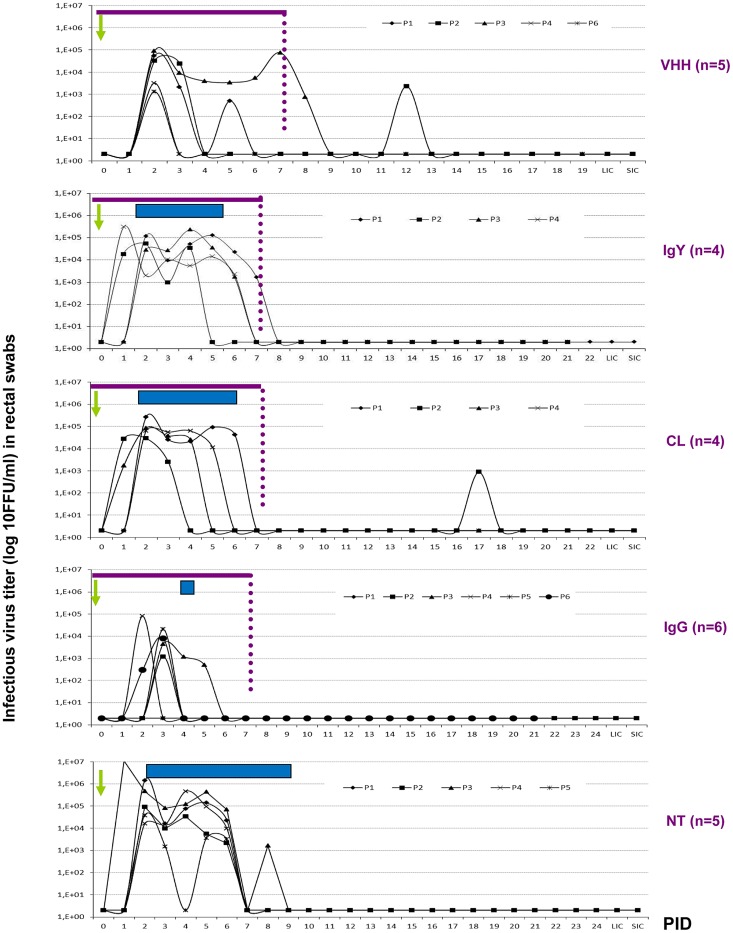
Mean titer of virus shed per day per pig (from CCIF assay) detected daily in rectal swab fluids of the experimental groups of piglets. All animals were orally inoculated at 72 h of age [0 post inoculation day (0 PID)], and euthanized at 21±3 PID. SIC: small intestinal contents; and LIC: large intestinal contents, were collected after euthanasia. Horizontal bars represent the mean days of diarrhea duration for each group. The arrow at 0 PID indicates the inoculation day. The thin line indicates the duration of the passive treatments.

The profiles of individual infectious virus shedding as determined by CCIF assay are depicted in Figure 1. Pigs in the VHH group showed a variable pattern of virus shedding, with some pigs shedding RVA for only one day and other pigs shedding virus for up to seven days. Only two pigs shed virus for one day after the end of the treatment, but in low titers. No diarrhea was observed in this group of pigs, indicative of asymptomatic virus shedding. In the IgG group, five pigs shed virus for up to three days during the administration of the treatment and there was no virus shedding after the end of the treatment. Although all the pigs in the CL group developed diarrhea and shed virus, one animal shed infectious virus after the treatment ended, but without diarrhea. The patterns of virus shedding in IgY, CL and NT groups were similar, with high titers of RVA particles shed for several days despite the administration of IgY or CL treatments (Figure 1 and Table 2).

### The oral administration of VHH nanoAbs did not affect the host immune response to Wa human RVA after experimental inoculation

**Figure 2:**
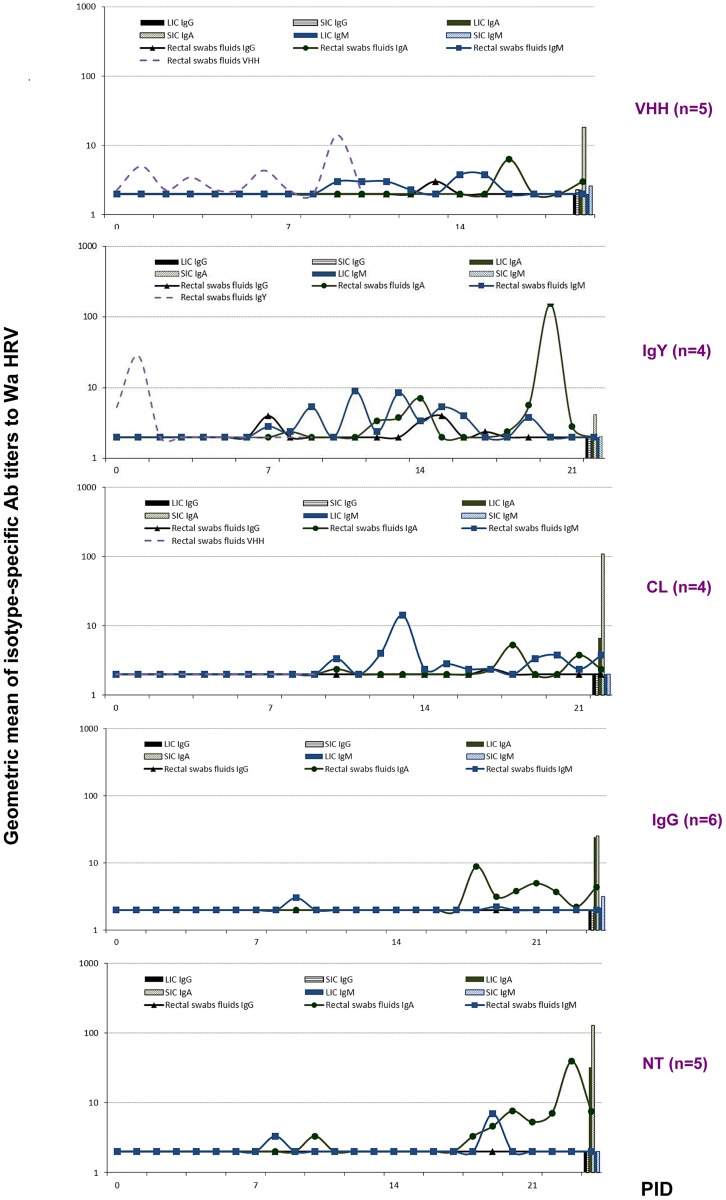
Geometric mean isotype-specific Ab titers (GMT) to Wa HRV per group per day (from ELISA assay) detected in rectal swab fluids of the experimental groups of piglets. All animals were orally inoculated at 72 h of age [0 post inoculation day (0 PID)], and euthanized at 21±3 PID. SIC: small intestinal contents; and LIC: large intestinal contents, were collected after euthanasia. The arrow at 0 PID indicates the inoculation day.

The presence of porcine coproAbs to Wa human RVA was evaluated daily in all the groups of pigs by isotype-specific ELISA (Figure 2). The IgM Abs were first detected at variable time points depending on the group of pigs and its presence was associated with virus clearance (Figure 1). The IgM response was always followed by IgA Ab responses. All the Ab titers detected were low and this may be explained by the fact that samples were diluted rectal swab fluids and not feces. In particular, IgA was the main isotype detected in rectal swab fluids and intestinal contents.

We further evaluated the persistence of the VHH, IgY and IgG Abs in rectal swab fluids after the treatments (Figure 2). The VHH nanoAbs administered were detected in rectal swab fluids from treated piglets by ELISA until up to two days after the end of the milk supplementation (PID 9), but always in low titers. The VP6-specific IgY Abs were also detected in rectal swab fluids, but only at the beginning of the milk diet supplementation. Although the supplemented Abs are indistinguishable from the host's immune response, no swine IgG Abs to RVA Wa were detected in rectal swab fluids from the IgG group of pigs during the treatment (Figure 2) suggesting that the treatment is being degraded in the intestinal tract.

**Figure 3:**
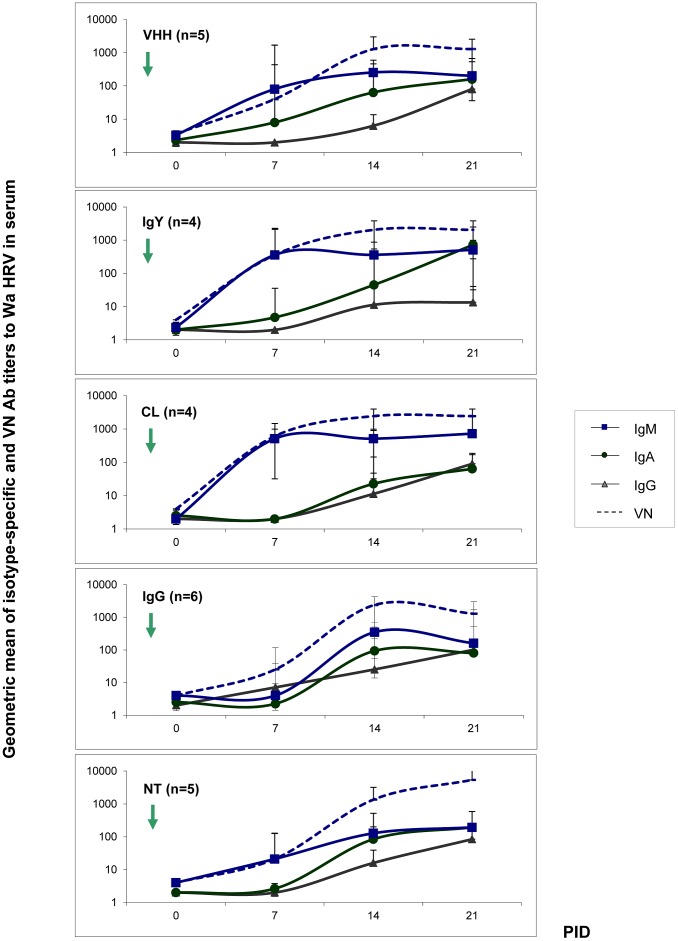
Wa HRV-specific porcine Abs in piglet's sera. The isotype-specific and VN Abs to Wa HRV in serum samples were determined weekly by ELISA and VN assay. The arrow indicates the experimental inoculation with Wa HRV (0 PID). PID: post inoculation days.

The porcine serum Ab isotype responses to Wa RVA are depicted in Figure 3. At PID 7, IgM was the main Ab isotype detected in pigs' sera and titers coincided with the VN activity detected. For all the experimental groups of pigs, except for the IgG group, these results were also in agreement with the coproAb responses previously described (Figure 2). Pigs in groups VHH, IgY and CL showed statistically higher titers of IgM Abs in serum at PID 7 (p = 0.0015) and PID 14 (p = 0.0033) than the IgG group; the NT group had intermediate titers at this experimental time point (PID 7). No other significant differences in porcine Ab titers to Wa RVA were detected in sera. There was a progressive increase in VN Ab titers over time during the experiment in animals from all groups, with the highest titers detected at PID 21, together with the presence of IgA and IgG Abs to Wa RVA (Figure 3).

No heterologous Abs derived from the treatments (Abs to VHH and IgY) were detected in serum samples from treated piglets at PID 0, 7, 14 and 21 by ELISA (data not shown).

### Ab Secreting Cell responses to Wa RVA infection were detected in all the tissues studied from all the experimental groups at PID 21

**Figure 4:**
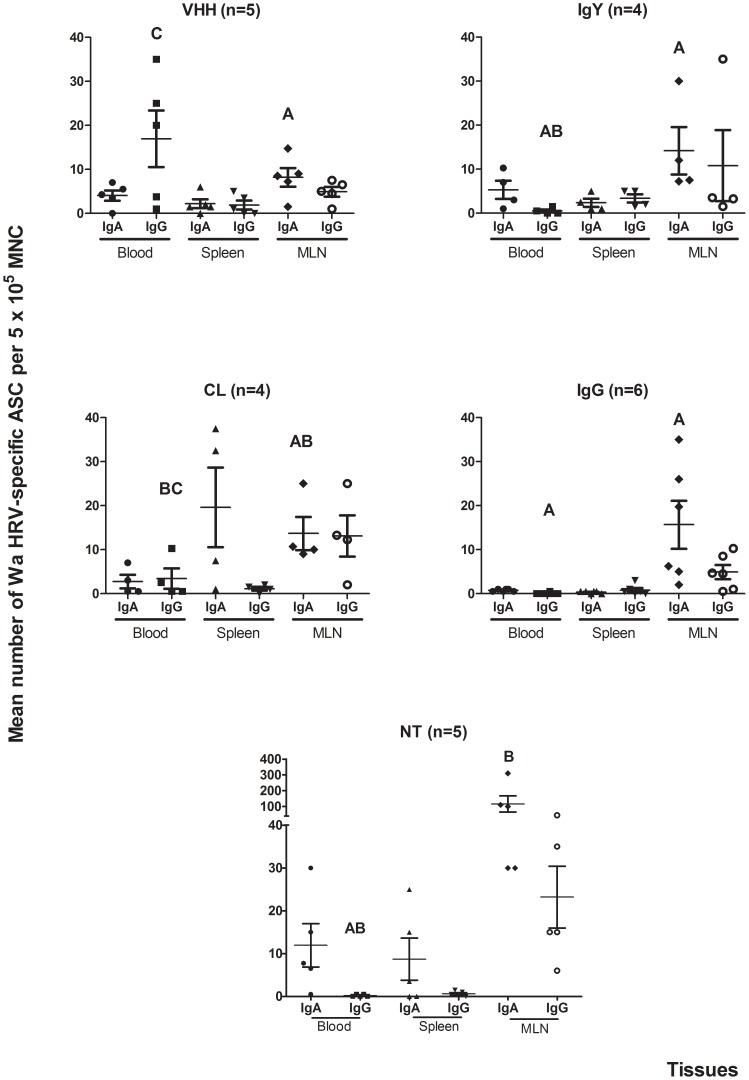
Numbers of Wa HRV-specific ASC per 5×10^5^ MNC obtained from systemic lymphoid tissues (blood and spleen) and MLN draining the small and large intestine at 21 PID. When comparing ASC numbers of the same isotype among treatment groups, different letters indicate a significant difference (Kruskal-Wallis rank sum test, p<0.05). n = number of piglets in each group. The IgM ASC response was not included as no IgM ASC were detected in most of the groups of pigs.

**Figure 5:**
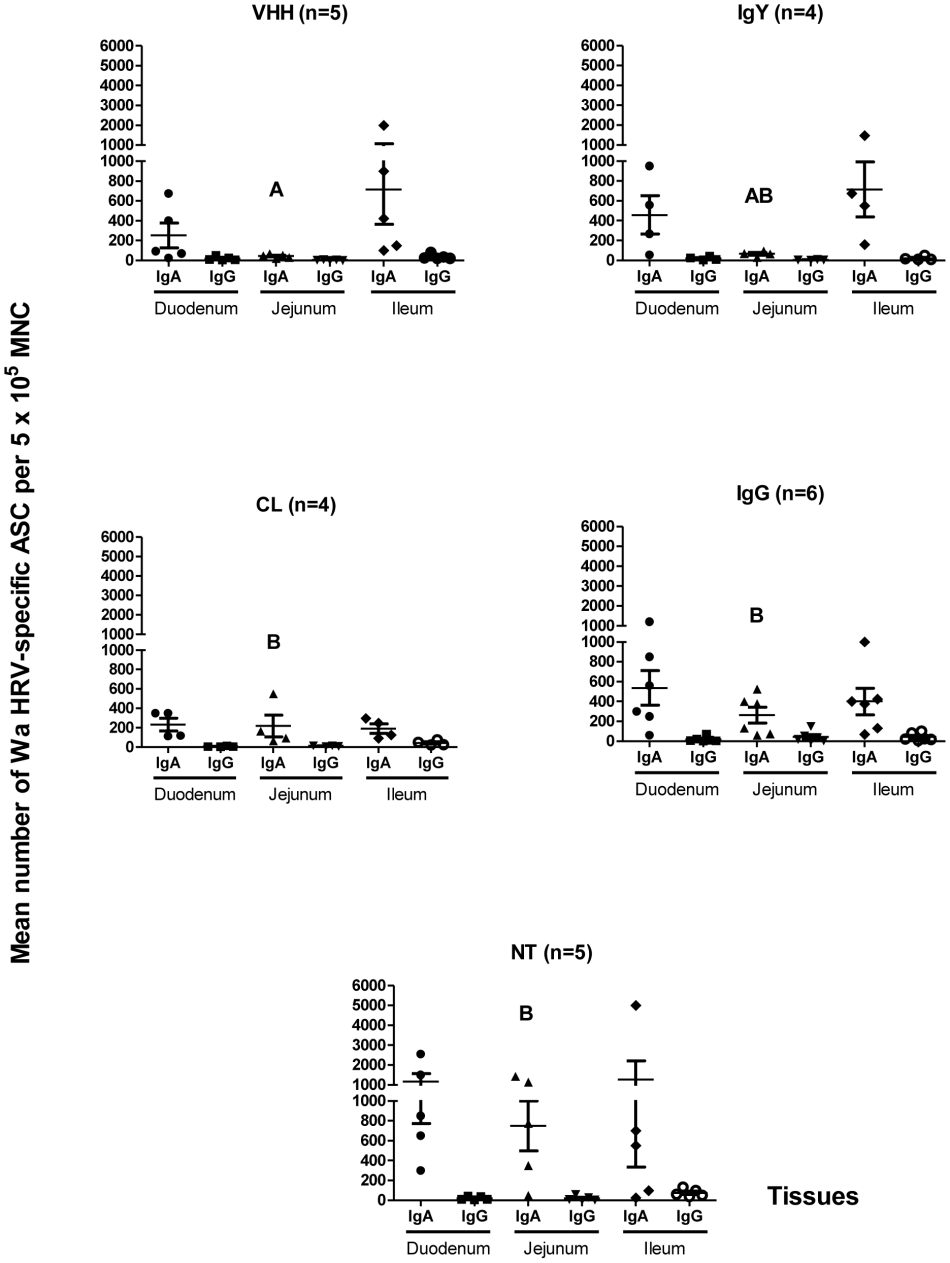
Numbers of Wa HRV-specific ASC per 5×10^5^ MNC obtained from gut-associated lymphoid tissues (Duodenum, Jejunum, Ileum) 21 PID. When comparing ASC numbers of the same isotype among treatment groups, different letters indicate a significant difference (Kruskal-Wallis rank sum test, p<0.05). n = number of piglets in each group. The IgM ASC response was not included as no IgM ASC were detected in most of the groups of pigs.

The Ab Secreting Cell (ASC) responses to RVA in systemic lymphoid tissues and gastrointestinal lymphoid tissues (GALT) at PID 21 were evaluated by ELISPOT assay and (Figures 4 and 5, respectively). Results for IgM ASCs in tissues at PID 21 are not shown since few of them were detected. There were statistically higher IgG ASC numbers in blood from the VHH group compared with the IgG group, while the CL, IgY and NT groups showed intermediate numbers (p = 0.003) (Figure 4). The numbers of IgA ASC were significantly higher in mesenteric lymph nodes (MLN) from the NT group than in the groups receiving human RVA-specific Abs as treatments (VHH, IgY and IgG groups) (p = 0.026). No further significant differences were observed in ASC numbers in systemic lymphoid tissues and MLN between groups (Figure 4).

Different numbers of ASC to Wa RVA at PID 21 in the GALT were detected in all the intestinal tissues studied (Figure 5). At PID 21, IgA was the main ASC isotype detected in the GALT from all groups of pigs, followed by IgG. The ASC numbers were statistically similar among groups. However, the IgA ASC numbers from jejunum were significantly lower in the VHH group compared with all other groups of pigs (p = 0.019). A trend towards lower numbers of ASC was observed in the VHH group compared with the NT group, but no other significant differences were detected (Figure 5).

### Nanobodies-specific immune responses were not detected in VHH treated piglets during the study period

**Figure 6:**
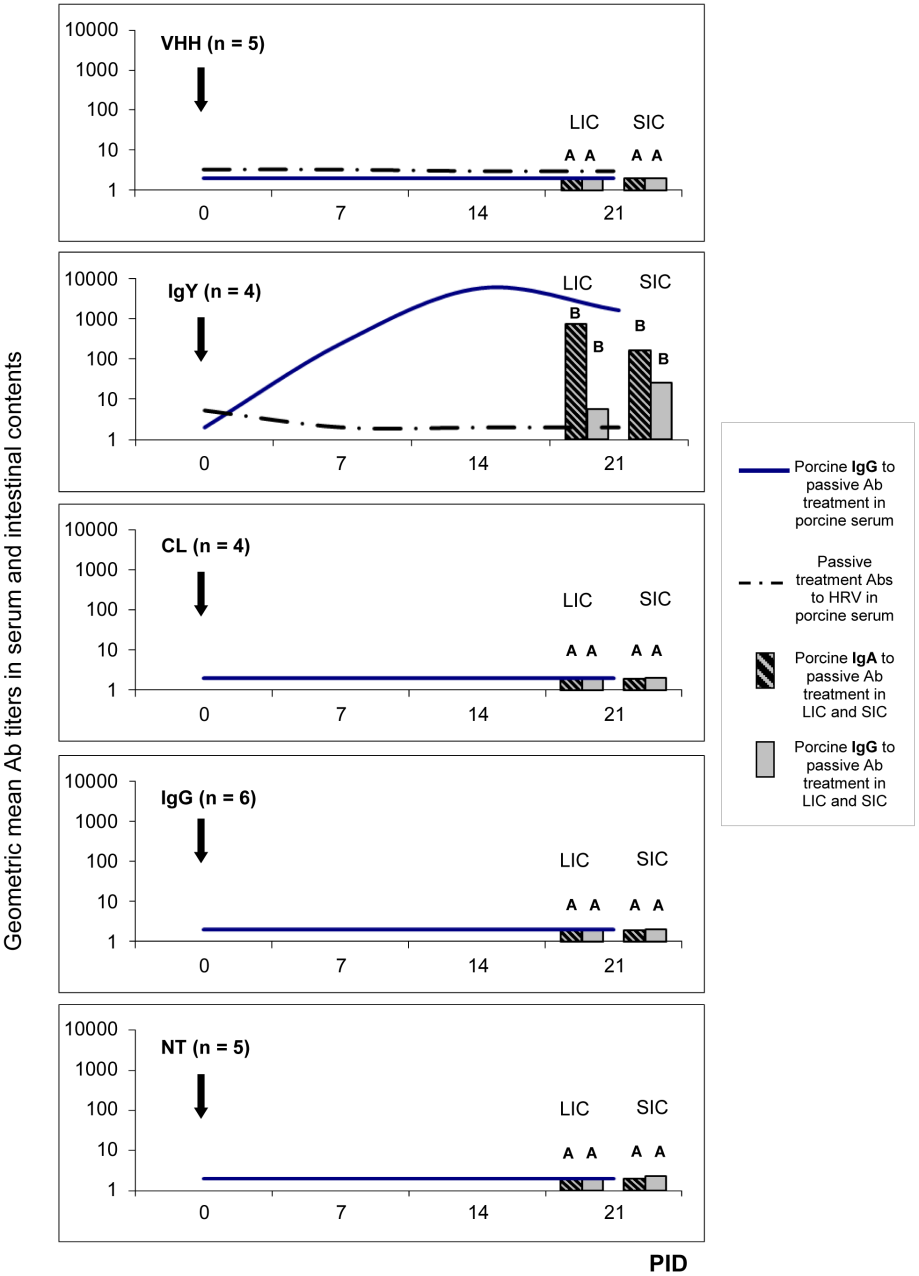
Immune responses to the passive treatments were determined in sera and intestinal contents of treated piglets. Continuous lines represent geometric mean of porcine IgG Ab titers (GMT) to the corresponding treatment administered (VHH, IgY or IgG Abs) in serum samples per group every 7 days. Dashed lines represent ELISA Ab titer of passive Abs to Wa HRV (VHH, IgY or IgG Abs) in rectal swabs every 7 days. The bars at PID 21 represent porcine IgG and IgA Abs to passive treatment in SIC: small intestinal contents and LIC: large intestinal contents collected after euthanasia. All animals were orally inoculated at 72 h of age [0 post inoculation day (0 PID)], and euthanized at PID 21±3. The arrow at PID 0 indicates the inoculation day. Different letters indicate significant differences between groups (p<0.05). In the case of groups CL, IgG and NT the results represent porcine IgG Ab titers to chicken IgY in serum but a similar response was observed against VHH nanoAbs (data not shown).

The presence of a nanobody-specific immune response in VHH and IgY treated animals was determined by ELISA in sera and intestinal contents of treated piglets (Figure 6). The IgG Ab levels were determined in serum samples weekly. In the intestinal contents, IgA Ab responses were determined together with IgG Abs to VHH nanoAbs at PID 21. In spite of the presence of low titers of orally administered VHH nanoAbs in rectal swab fluids from the VHH group, the treated piglets did not develop specific Ab responses to the VHH nanoAbs neither in serum nor in the intestinal contents by PID 21 (Figure 6). Further analysis of rectal swab fluids also failed to detect porcine Abs to the VHH nanoAb (data not shown).

In contrast, piglets fed with milk supplemented with IgY Abs to VP6 developed a humoral Ab response to the IgY Ab, (Figure 6). Piglets in the IgY group developed serum IgG Abs to IgY at PID 7 (9 days after starting the treatment), with a peak at PID 14. The IgA and IgG Abs to chicken IgY were also observed in the intestinal contents at PID 21 (Figure 6), but not in rectal swab fluids (data not shown).

As expected for the piglets that did not receive heterologous immunoglobulins, no IgG/IgA Abs to IgY or VHH were detected in CL, IgG and NT piglets in serum, rectal swab fluids or intestinal contents (Figure 6 and data not shown). Swine Abs to porcine IgG were not tested, as an immune response of the host to homologous Abs is not expected.

### VHH 3B2 showed broad neutralization activity against several RVA genotypes *in vitro*

To further characterize the ability of VHH nanoAbs to neutralize RVA, a virus neutralization assay was performed using several RVA strains. The RVA strains were selected considering their G-, P- and I-genotypes in order to include as many genotypes circulating in humans as possible (Table 3).

**Table 3:** *In vitro* RVA fluorescent focus reduction assay using VHH antibodies.

RVA strain	Neutralizing VHH concentration (µg VHH/ml)	
	3B2 VHH clone	Non-related VHH clone
**Wa** *(G1 P[8] I1)*	0.06	Neg
**DS1** *(G2 P[4] I2)*	0.24	Neg
**SA11** *(G3 P[1] I2 )*	15.63	Neg
**Gottfried** *(G4 P[6] I1)*	0.24	Neg
**ST3** *(G4 P[6] I1)*	0.98	Neg
**H1** *(G5 P[7] I5 )*	0.98	Neg
**69M** *(G8 P[10] I2)*	0.98	Neg
**F45** *(G9 P[8] I?)*	0.06	Neg
**Arg720** *(G12 P[9] I?)*	3.91	Neg

A fourfold dilution of each VHH clone (3B2 VHH, derived from llamas vaccinated with VP6 from RVA strain C486 -G6P[1]I?-,or non-related VHH nanoAb) was mixed with an equal volume of RVA containing 100 focus forming units (FFU) of each strain. The numbers represent the minimum VHH concentration that reduced >80% of the number of fluorescent focus forming units (FFU) of each RVA strain. Neg = no neutralizing activity was observed at the highest concentration tested (62.50 µg VHH/ml). The G-type, P-type and I-type of RVA strains is clarified in brackets next to each strain name (according to [91]).

VHH nanoAb 3B2 was able to neutralize all the RVA strains tested at different concentrations, ranging from 15.63 µg VHH/ml for SA11 strain to 0.06 µg VHH/ml for Wa and F45 strains. As a control, non-related VHH nanoAb was not reactive against any of the RVA strains tested (Table 3).

## Discussion

The VP6 constitutes the inner capsid of rotavirus and it is the most abundant and immunodominant viral protein. The Abs directed to VP6 are highly cross-reactive among RVA so VP6 immunization could potentially provide heterotypic protection [62]. The main objective of the present study was to determine the efficacy of the oral administration of G6P[1] VP6-specific VHH nanoAbs against Wa G1P[8]I1 human RVA-induced diarrhea in a Gn pig model of infection and disease. The 3B2 VHH clone protected all treated animals from RVA-induced diarrhea when administered daily as milk supplement at a final Wa RVA ELISA Ab titer of 4096 (VN titer of 256) during nine consecutive days. The protection observed was comparable to that in pigs treated with porcine IgG Abs to Wa RVA at the same ELISA and VN titers. This latter group of pigs was considered the positive control group, simulating the protection conferred by homologous Abs [63], [64]. The presence of diarrhea in 2 out of 4 animals from the IgG group suggests that this passive treatment with homologous Abs is not completely capable of preventing RVA-induced diarrhea against the challenge dose administered. However, the homologous treatment still conferred a great degree of protection, highlighting the importance of passive maternal Abs in newborns. These results reinforce the importance of the use of RVA vaccines (to induce active immunity) but also state the precedent that additional RVA-specific strategies might be useful to control and prevent RVA diarrhea in human infants.

Passive or active protection against RVA diarrhea is the most desirable outcome of any treatment or vaccination and can be achieved even when continued viral shedding is detected in stools. Moreover, the asymptomatic infection allows development of active immunity (as noted in our study) to prevent subsequent natural RVA infections. In our study, passive treatment with homologous porcine IgG Abs also provided a high degree of protection against virus shedding. However, the production of large amounts of polyclonal human IgG Abs specific to RVA needed for children would be difficult to implement and Abs would be mostly RVA strain-specific. We investigated a more universal treatment against a wide range of RVA strains applicable for neonatal humans or animals, based on VHH technology, which is easy to scale up and presents distinctive advantageous properties over traditional antibodies.

The 3B2 VHH clone expressed in *E. coli* was previously characterized and showed a broad neutralizing activity against RVA *in vitro*, independently of the RVA serotype [54]. Remarkably, this VHH was developed against the VP6 of bovine C486 (G6P[1]I?) RVA strain, but it showed broad neutralization activity *in vitro* against RVA of heterologous serotypes and species of origin including Wa HRV [54]. Furthermore, this result was confirmed by *in vivo* partial passive protection against bovine RVA strain C486 and murine RVA strain EC_W_ (G16P[16]I7) at 48 h post challenge in a neonatal mouse model using 1 mg/ml (22,2 mg/kg/day) of recombinant VHH administered once a day for 5 consecutive days [54]. The 3B2 VHH clone expressed in insect cell cultures had the same therapeutic properties as the one expressed in *E. coli* in the neonatal mouse model [55]. In the present study, 3B2 VHH neutralized virus infection *in vitro* from several RVA strains and this seemed to be independent of the G-, P- and I-genotypes of RVA strains tested. The results obtained *in vitro* demonstrate that the 3B2 VHH nanoAb inhibits infection by relevant RVA strains circulating in humans worldwide, including not only Wa (G1P[8]I1), but also DS1 (G2P[4]I2), 69M (G8P[10]I2), F45 (G9P[8]I?) and Arg720 (G12P[9]I?) among others (Table 3). In addition, a high *in vivo* protection rate was observed in this study against VirWa human RVA inoculation in Gn pigs fed with 22 mg of VHH nanoAbs per 210 ml of milk (0.1 mg/ml of milk, 44 mg/pig/day or 20–40 mg/kg/day).

VHH nanoAbs protected against RVA-induced diarrhea in all the animals tested, even when all the animals shed virus infective particles. Since VHHs are recombinant monoclonal Abs, the possibility of VP6 escape mutant selection should be considered. Further studies are underway to determine if VHH nanoAbs promote VP6 escape mutants *in vitro* and *in vivo*. This information will help us better understand how the VHH nanoAbs inhibit RVA infection.

Chicken egg yolk IgY Abs to G6P[1] VP6 were also used as a control for treatment with heterologous conventional Abs. This treatment was not protective against RVA diarrhea or virus shedding in Gn pigs. These results obtained in gn pigs, an animal model that closely resembles RVA pathogenicity in human infants, are in agreement with the lack of protection observed in a mice model using other conventional Abs directed to VP6 protein [47], [48]. Of note, the avian IgY Abs were obtained from VP6-vaccinated hens with pre-existing VN activity to Wa RVA. Avian rotavirus is endemic in hens [11] and it has been reported that mammalian and avian RVAs have a low degree of nucleotide sequence identity for all eleven genome segments [39], [65]. This may explain the presence of only low pre-existing VN activity to RVA in the chickens due to cross reactivity.

Serum, coproAbs, and ASC responses were tested to assess the development of the pigs' immune responses to Wa RVA. The highest ASC responses to Wa RVA were in the GALT at PID 21, with lower ASC numbers in systemic lymphoid tissues. Our results indicate that pigs receiving passive Ab treatments (IgG, IgY or VHH) had the same isotype profile of IgA ASC but of lower magnitude than piglets in NT group, suggesting some interference effect. A short delay in the development of the immune response and a lower number of ASC were also evident in the group of pigs treated with VHH (in particular, reduced IgA ASC in jejunum). However, the magnitude of the Ab responses developed was potent enough to prevent Wa RVA diarrhea after a second exposure to the virus, in agreement with previous reports [66]. However, it is unclear if the passive treatment with VHH nanoAbs affected the development of the pigs' immune response to RVA infection. The absence of diarrhea, and thus a lower level of viral replication, probably affects the development of a stronger immune response.

The administration of control larvae as passive treatment (CL) failed to prevent RVA-associated diarrhea and did not significantly modify piglets' immune responses to Wa human RVA, but showed a trend toward less severe diarrhea, virus shedding and lower number of ASC compared to the NT group. An explanation for this phenomenon is not clear and may be related to biological factors in the harvested *T. ni* larvae that may have an adjuvant or immunopotentiating effect on innate immunity in the Gn pigs. It has been described that baculovirus could elicit antiviral activity in mammalian cells, both *in vitro* and *in vivo*, but this hypotheses should be further explored [67]. However, the control larvae group trend to reduce virus shedding was not enough to statistically modify the diarrhea-associated parameters, as it was not significantly different from NT control group.

A critical point to be considered is the host's immune response to the passive treatment with heterologous Abs, as it has serious health implications such as the development of allergy and other hypersensitivity reactions. In this study, a heterologous neutralizing VHH nanoAb was examined as a prevention strategy against human RVA in an animal model that closely mimics the intestinal physiology of human infants. VHH-treated piglets did not develop detectable humoral Ab responses against the passive VHH treatment (in serum, rectal swab fluids, or intestinal contents) by the methodology used. In contrast, piglets treated with IgY Abs to VP6 developed a humoral Ab response to the passive IgY treatment, characterized by the presence of IgG Abs to IgY in serum and both IgG and IgA Abs to IgY in the intestinal contents. Equally important, no heterologous Abs derived from the VHH or IgY passive treatment were detected in sera of treated animals, which suggests that there was no transmission of VHH or IgY Abs from the gut lumen to the blood. It is well documented that under physiological, non-inflamed conditions, dendritic cells migrate from mucosa to present antigens in the lymph nodes [68]. As a result, dietary antigens are presented in association with signals that drive a suppressive immune response. It has been reported that IgY is highly immunogenic as it has been used as a model antigen for testing mucosal adjuvants, which might explain the presence of IgG anti IgY in sera of treated animals [69], [70]. On the contrary, the oral administration of VHH nanoAbs might induce a tolerant state, preventing the host from eliciting an anti-VHH immune response, but further studies must be conducted to confirm this statement.

It has been previously reported that passive VHH nanoAbs to VP6 with similar VN activity were protective against RVA *in vivo* in mice [71]–[75]. Briefly, Van der Vaart *et al.* produced VHH nanoAbs to a G3 Rhesus-monkey RV (RRV) strain in yeast. Daily oral administration of 50–100 µg/pup (20–40 mg/kg/day) during five days reduced the morbidity of RRV-induced diarrhea in mice, but it was unclear whether the VHH nanoAbs were effective against other rotavirus strains [73]. Pant *et al.* also produced VHH nanoAbs to G3 RRV in *Lactobacillus paracasei*, in both secreted and cell surface–anchored forms. Lactobacilli expressing membrane anchored nanoAbs showed neutralizing activity *in vitro*. The prophylactic use of those VHH nanoAb during 5 days reduced the frequency, duration and severity of diarrhea in mouse pups after oral inoculation with RRV [72], [75]. Recently, Aladin *et al.* further characterized the same VHH clones (ARP1 and ARP3) and reported *in vitro* neutralizing activity against several RVA strains including human RVA strains Wa, DS1, 69M, F45 and ST3, but failed to neutralize SA11 and one of the RRV strains tested. Although these VHH nanoAbs were developed against complete RRV, ARP1 and ARP3 are also directed to VP6 as evidenced by western blot analysis. [71] In contrast, ARP1 and ARP3 recognize a lineal epitope of VP6 unlike 3B2 VHH that is directed to a conformational epitope [54], [71]. Collectively, the evidence obtained from Aladin *et al.* together with our results suggest that the ability of VHH nanoAbs to inhibit *in vitro* virus replication does not correlate with a specific VP6 genotype as several RVA strains with different G-, P- and I-genotypes were neutralized by VHHs. However, 3B2 VHH appears to be much more efficient in inhibiting strain-specific RVA replication than ARP1 and ARP2 VHHs [71], as lower amounts of 3B2 VHH were needed to neutralize Wa, DS1, ST3, F45 and 69M RVA infection *in vitro*. This assumption should be confirmed testing the VHH nanoAbs (3B2, ARP1 and ARP3) under the same conditions.

The VHH nanoAbs were the first Abs to demonstrate extracellular neutralizing activity. The mechanism by which VHH nanoAbs inhibit virus replication is still unclear and several studies are in progress in our laboratory to elucidate it. With this in mind, Feng *et al.* showed that the attachment of a VP6-specific IgA monoclonal Ab to VP6 introduced a conformational change in the VP6 trimer, rendering the particle transcriptionally incompetent and preventing the elongation of initiated transcripts [45]. Since monoclonal Abs to VP6 are capable of inhibiting RVA transcription [76], it reinforces the idea that VP6 IgA Abs confer protection *in vivo* by inhibiting viral transcription at the start of the intracellular phase of the viral replication cycle. It is widely known that the VP6 protein plays a key role in the organization of the virion, because it is in direct contact with the inner and the outer protein shells. Likewise, VP6 is the physical adaptor between two biological functions critical for RVA: cell entry (related to the virus outer layer) and genomic RNA packaging (related to the inner layer) [11], [77]. Although the VP6 protein is only exposed in double-layered particles, VHH nanoAbs of smaller size than conventional Abs, could penetrate the outer shell, bind VP6, and block decapsidation, inhibiting the early steps of virus replication. An improved structural understanding of the common features of the different RVA strains neutralized by VHH nanoAbs will shed light into this mechanism.

Finally, our results indicate that the VHH clone 3B2 is a recombinant monoclonal nanoAb that should be further explored as a potential treatment of RVA-associated diarrhea, as it showed 100% efficacy against Wa G1P[8]I1 RVA-induced diarrhea *in vivo*, and a broad neutralizing activity against several RVA strains circulating in humans worldwide *in vitro* [54]. This VHH nanoAb would also have the advantage of producing and administering a single antibody in contrast to a cocktail of different conventional Abs directed to the common G-P RVA strains circulating in human populations. Additionally, it could be scaled-up to develop pediatric medications or functional foods like infant milk formulas to control RVA diarrhea. Producing VHHs using an expression system with high production efficiency or downstream processing is crucial, since the commercial application of this treatment will greatly depend on the VHH nanoAb production costs. Additional studies will be needed to determine the safety and efficacy of VHHs against different RVA wild type strains as well as the stability and administration regime of a treatment. In summary, our findings show the potential value of broad neutralizing VP6-specific VHH nanoAbs as a treatment that could complement (but not replace) the use of serotype-specific RVA vaccines. This study represents a proof of principle of the efficacy of VHH oral therapy to prevent RVA diarrhea. This type of treatment has great potential to be implemented in developing countries, where RVA mortality is high and current vaccines seem less efficacious, and also to be administered to prematurely born or immunodeficient children worldwide [25], [78].

## Materials and Methods

### Ethics statement

This study was carried out in strict accordance with the recommendations by the Public Health Service Policy, United States Department of Agriculture Regulations, the National Research Council's Guide for the Care and Use of Laboratory Animals, and the Federation of Animal Science Societies' Guide for the Care and Use of Agricultural Animals in Agricultural Research and Teaching, and all relevant institutional, state, and federal regulations and policies regarding animal care and use at The Ohio State University. The study protocol was approved by the Committee on the Ethics of Animal Experiments of The Ohio State University (Protocol Number: 2010A00000088). All the pigs were maintained, samples collected, and then euthanized, and all efforts were made to minimize the suffering of animals.

Oral inoculation of neonatal pigs with Wa RVA caused transient diarrhea of variable severity from which piglets recovered spontaneously within a few days. The euthanasia was performed by electrocution following anesthesia. For this particular study, we used the lowest number of pigs previously shown to permit detection of statistical significances among treatments considering welfare principles. A statistician verified the minimum number of pigs required per treatment group before starting the experiments.

### Preparation of Ab pools

#### VP6-specific recombinant VHH nanoAbs

The recombinant baculovirus expressing 3B2 or a non-related VHH clone was constructed as previously described [55]. We incorporated a His-tag sequence at Carboxy-terminus of the protein to facilitate the VHH purification. The *T. ni* larvae were intraemocelically injected with 5 µl of recombinant baculovirus inoculum containing 5×10^4^ plaque forming units (PFU) and were kept in growth chambers at 28°C in specifically designed rearing insect modules. Infected larvae were collected after 72 h post infection (hpi) and immediately frozen at −70°C. A comparable weight of mock-infected control larvae was equally processed. Larvae were lyophilized overnight in a Telstar Cryodos freeze-dryer (Telstar, Madrid, Spain), and stored at 4°C until used. Lyophilized insect material (18 g of freeze-dried insect larvae containing about 400 mg of VHH and the same amount of control larvae) was crushed in sterile plastic bags by a blender to obtain a powder, which was kept at 4°C until use. To recover the recombinant VHH, the larvae powder contained in BagFilter S bags (Interscience, Saint Nom, France) was reconstituted in 360 ml per batch of sterile phosphate-buffered saline (PBS), pH 7.4 as extraction buffer. The resulting suspension was filtered through the bag filter paper (porosity of the filter <250 microns), recovered and centrifuged at 10,000×g for 15 min, and the pellet was discarded. The supernatants were sterilized by filtration (0.22-µm-pore-size membrane filter; Millipore, Billerica, USA), divided in 10 ml aliquots and stored at −20°C until used. The 3B2 VHH nanoAbs recovered from the larvae powder batch had an ELISA Ab titer against Wa RVA of 1,048,576 and virus neutralization (VN) titer of 65,536. The preparation from mock-infected larvae tested negative in both assays (<4). The 10 ml aliquots of the corresponding preparation were used to supplement 210 ml of Ab free commercial bovine milk. For the 3B2 VHH supplemented milk (VHH group of pigs), each 10 ml aliquot contained 22 mg of VHH nanoAbs and had a Wa RVA ELISA Ab titer of 4096 and a VN titer of 256. The milk supplemented with mock infected larvae (Control Larvae –CL- group of pigs), had a Wa RVA ELISA Ab titer of <4 and a VN titer of <4, as detailed in Table 1.

#### VP6-specific IgY Abs from egg yolk

The egg yolk pools used in this study were obtained from immunized hens. Eight-week old Lohmann Brown Classic laying hens were housed in cages specially designed for this purpose in groups of two animals per cage following animal welfare recommendations [79], [80]. Room temperature, relative humidity and light/dark cycles were controlled. Hens were fed laying hen diet and provided water *ad libitum*, and eggs were collected daily starting at the first immunization. To generate VP6-specific IgY Abs from egg yolk, ten hens were hyperimmunized with recombinant VP6 from RVA as previously described [53]. Serum samples and egg yolks were collected, and IgY Ab titer to Wa RVA was determined by ELISA. Pools of egg yolks from the immunized hens were prepared weekly and were diluted in distilled water at a ratio of 1∶3, and freeze-thawed to separate the emulsion. The Abs were precipitated following an ammonium sulphate precipitation protocol as previously described [53]. The pellet obtained was resuspended in PBS pH 7.4 at a 1∶10 ratio of the original volume of the egg yolk pool and dialyzed against the same buffer, sterilized by filtration (0.22-µm-pore-size membrane filter; Millipore), divided in 10 ml aliquots and stored at −20°C until used. The final volumes recovered were slightly different from the initial ones after dialysis. The IgY Ab titer to Wa RVA was determined by ELISA and VN assays. The 210 ml of sterile bovine milk supplemented with 10 ml of IgY preparation (IgY group of pigs) had a final ELISA IgY Ab titer to Wa RVA of 4096 and a VN titer of 64 (Table 1).

#### Wa RVA IgG Abs from immune sow serum

RVA-seropositive sows (n = 2) received five doses of attenuated Wa RVA (∼10^7^ FFU/dose) intramuscularly at 2-week intervals, with the first dose in Freund Complete Adjuvant (FCA), followed by three doses in Freund Incomplete Adjuvant (FIA) and the last one without any adjuvant. A week after the last immunization, serum was collected and pooled as previously described [53]. The Abs were precipitated following the same ammonium sulphate protocol used for IgY Abs and then filtered (0.22-µm-pore-size membrane filter; Millipore), divided in 10 ml aliquots and stored at −20°C until used. Purified IgG Abs from sow serum served as the source of positive homologous IgG Abs to Wa RVA. Each 210 ml portion of milk supplemented with 10 ml of IgG preparation (IgG group of pigs) had a final ELISA IgG Ab titer to Wa RVA of 4096 and a VN titer of 256 (Table 1).

### Virus

The intestinal contents of RVA Wa G1, P1A [8] infected Gn pigs were diluted in minimal essential medium (MEM; Invitrogen, Carlsbad, USA), and used for virus inoculation as described [64]. Attenuated Wa HRV (cell culture adapted) was propagated in monkey kidney (MA-104) cells for use in sow hyperimmunization, enzyme-linked immunospot assay (ELISPOT), ELISA and VN assays. The RVA strains DS1 (G2P[4]I2), SA11 (G3P[1]I2), Gottfried (G4P[6]I1), ST3 (G4P[6]I1), H1 (G5P[7]I5), 69M (G8P[10]I2), F45 (G9P[8]I?) and Arg720 (G12P[9]I?) were propagated in monkey kidney (MA-104) cells for use in VN assays.

### Continuous cell lines

The MA-104 cell line of fetal rhesus monkey kidney cells (passage 36) was originally purchased from Microbiological Associates (now BioWhittaker, Radnor, USA). The Sf9 cell line, a clonal isolate derived from *Spodoptera frugiperda* (Fall Armyworm), was obtained from Invitrogen and cultured in Grace's insect media (GIBCO, Carlsbad, USA) supplemented with 10% fetal bovine serum (FBS, GIBCO), 3% non-essential amino acids (Invitrogen), 1% nystatin, streptomycin and penicillin and 20 µg/ml gentamycin (Invitrogen). Sf9 cells were grown at 27°C and MA-104 cells at 37°C in a 5% CO_2_ –air atmosphere.

### Gnotobiotic pigs: Experimental design, virus inoculation, clinical observations and sample collection

#### Gnotobiotic pigs

the Gn pigs were derived by hysterectomy of near-term sows and maintained in isolator units as previously described [81], [82]. Pigs were allocated to one of five groups as detailed in Table 1. The ELISA Ab titer to Wa RVA was used as the adjusting parameter to compare the three different Ab passive treatments used: monoclonal VP6-specific llama-derived 3B2 VHH nanoAbs; VP6-specific chicken egg yolk IgY Abs; and Wa RVA-specific homologous porcine IgG Abs (VHH, IgY and IgG groups respectively). During the first 24 h of life, piglets received commercial sterilized bovine milk (RVA Ab free) for human consumption (Parmalat, St. Paul, USA) *ad libitum*. From the second day of life on, the milk diet consisted of 210 ml of RVA Ab free commercial milk supplemented with the corresponding volume of passive treatment pools, as described in Table 1, twice a day (9 days of passive treatment). A No Treatment (NT) group of pigs received no Ab treatment and was fed only the commercial milk. The passive treatment administration started after the main period of gut closure, at approximately 36 h of age, in order to reduce systemic Ab absorption [83], [84]. From the end of the passive treatments onwards, all pigs were fed only the Ab free milk twice a day until the end of the experiment at 21 post inoculation days (PID).

#### Pig virus inoculation, clinical observations and sample collection

At 72 h of age, pigs in all groups received 5 ml of 100 mM sodium bicarbonate (pH 9.5, to neutralize gastric acidity), followed by 10^6.7^ FFU of RVA Wa in 5 ml of MEM (Invitrogen) [64]. The 50% infectious dose (ID_50_) and diarrhea dose (DD_50_) of RVA Wa was determined previously in Gn pigs as ≤1 FFU [85], [86]. After inoculation, piglets were examined daily for diarrhea and virus shedding from PID 0 to 21. To estimate the severity of the diarrhea, fecal consistency was scored a blinded manner by the same individual throughout as follows: 0: normal; 1: pasty; 2: semi-liquid; 3: liquid. A score equal or greater than 2 was considered diarrhea. Prior and after RVA Wa inoculation, rectal swabs were collected daily to assess virus shedding. Briefly, swabs were resuspended in 8 ml MEM (Invitrogen), corresponding to a 1∶25 dilution. All samples were tested by ELISA for assessment of antigen shedding and by cell culture immunofluorescence (CCIF) assay for detection of infectious virus shedding. Serum samples were collected before the beginning of the passive treatments (within 24 h after birth), at virus inoculation, and then weekly (PID 7, 14 and 21). Serum Ab levels to Wa RVA were measured by isotype-specific ELISA and VN assays. The presence of coproantibodies (coproAb) was also assessed by ELISA. At PID 21±3, the animals were euthanized to study the Ab secreting cells responses to Wa RVA. The primary RVA-specific Ab secreting cells (ASC) were quantified by ELISPOT assay in the following gut-associated lymphoid tissues (GALT): duodenum, jejunum and ileum lamina propria (LP), mesenteric lymph nodes (MLN); and in the systemic lymphoid tissue spleen and the blood (PBL). Large (LIC) and small (SIC) intestinal contents from all the piglets were collected at necropsy for coproAb detection by ELISA [63], [64].

### RVA antigen and viral detection

Human Wa RVA shedding was detected in rectal swab fluids using an antigen capture ELISA as described previously [63], [64], [87]. Virus infectious titer was assessed by CCIF assay as described previously [52]. Fluorescent cells were counted using a fluorescence microscope and titers were expressed as the number of fluorescent focus forming units per ml (FFU/ml). The area under the virus shedding (FFU/ml by CCIF) curve through time (PID) (AUC) was calculated using statistical software and expressed as FFU/ml*day. First, the AUC for each piglet was determined and then the average AUC was calculated for the group.

### Wa RVA-specific recombinant VHH nanoAb ELISA

The VHH nanoAb titers to Wa RVA were quantified in the VP6-specific VHH nanoAb pool, supplemented milk, pig sera, rectal swab fluids, LIC and SIC. Briefly, 96 well ELISA plates (Maxisorp, NUNC, Roskilde, Denmark) were coated with 1∶10,000 dilution of RVA-specific guinea pig polyclonal serum at 37°C during 1 h and then incubated with 10% nonfat milk in PBS-Tween 0.05% for blocking of non-specific activity. The supernatants of semi-purified attenuated (Att) Wa RVA-infected (10^5^ FFU/ml) MA-104 cell culture lysates or mock infected MA-104 cell lysates were then added, followed by serial four-fold dilutions of the samples. VHH were detected using a rabbit polyclonal anti-VHH serum (1∶7,000 dilution). This rabbit antiserum was made in house, vaccinating the animal with a cocktail of several VHH nanoAbs. The plates were later incubated with commercial HRP-labeled goat polyclonal Abs to rabbit IgG at a 1∶2,000 dilution (KPL, Kirkegaard & Perry Laboratories Inc., Gaithersburg, USA) at 37°C for 1 h. Hydrogen peroxide and 2,2′-azino-bis(3-ethylbenzothiazoline-6-sulphonic acid (ABTS) were used as substrate/chromogen system (KPL, Kirkegaard & Perry Laboratories Inc.). The cut off point for the assay was established as the mean plus three standard deviations of the optical density measured in virus-coated PBS wells (blank).

### Wa RVA-specific IgY Ab ELISA

The IgY Ab titers to Wa RVA were determined in hen sera, crude egg yolks, purified IgY pools, supplemented milks, pig sera, rectal swabs, LIC, and SIC by an indirect ELISA as described elsewhere [53]. The cut off point for the assay was established as the mean plus three standard deviations of the optical density measured in virus-coated PBS wells (blank).

### Porcine isotype-specific Ab to Wa RVA ELISA

The IgM, IgA and IgG Ab titers to Wa RVA were quantified in the Wa RVA porcine IgG Ab pool, pig sera, rectal swab fluids, LIC, and SIC. Specific Abs to Wa RVA were detected by an indirect ELISA using the reagents and protocol described previously [63], [64], [82]. The cut off point for the assay was established as mean plus three standard deviations of the optical density measured in virus-coated PBS wells (blank).

### Porcine IgG and IgA Ab ELISA to recombinant VHH

The IgG Ab titers to recombinant VHH were quantified in the pig sera, rectal swab contents, LIC, and SIC. The IgA Ab titers to recombinant VHH were quantified only in rectal swab contents, LIC, and SIC. Briefly, 96 well ELISA plates (Maxisorp, NUNC) were coated with 1 µg per well of purified bivalent VHH nanoAbs (provided by Algenex S.L., Madrid, Spain) and then incubated with 10% nonfat milk in PBS-Tween 0.05% for blocking of non-specific activity. Serial four-fold dilutions of porcine serum, rectal swab contents or intestinal content samples were incubated for 1 h at 37°C. The plates were later incubated with commercial HRP-labeled goat polyclonal Abs to porcine IgA at 1∶3000 dilution (AbD Serotec Inc., Raleigh, USA) or with commercial biotin-labeled goat polyclonal Abs to porcine IgG at a 1∶20,000 dilution (KPL, Kirkegaard & Perry Laboratories Inc.) for 1 h at 37°C. The wells that contained commercial anti-pig IgG Ab were later incubated with commercial HRP-labeled streptavidin (1∶10,000) (Sigma-Aldrich, Munich, Germany) for 1 h at 37°C. Hydrogen peroxide and ABTS were used as substrate/chromogen system (KPL, Kirkegaard & Perry Laboratories Inc.). This ELISA was also performed using a second set of reagents (HRP-labeled commercial anti-pig IgG Ab from KPL, Kirkegaard & Perry Laboratories Inc.) to confirm the results obtained. The cut off point for the assay was established as the mean plus three standard deviations of the optical density measured in virus-coated PBS wells (blank). The ELISA used to detect porcine Abs against VHH nanoAbs in the present study was analytically validated against positive control sera from animals systemically immunized with a pool of VHH nanoAbs. This assay was able to detect the presence of VHH -specific Abs in this samples that were then considered positive controls for each assay performed.

### Porcine IgG and IgA Ab ELISA to chicken IgY

The IgG Ab titers to chicken IgY were quantified in the pig sera, LIC, and SIC. The IgA Ab titers to IgY Ab were quantified only in LIC and SIC. Specific Abs to IgY Ab treatment were detected by an indirect ELISA using the reagents and protocol described previously [53]. The ELISA used in the present study to detect porcine Abs against IgY Ab was analytically validated against positive control sera from animals systemically immunized with IgY Abs. This assay was able to detect the presence of IgY-specific Abs in this samples that were then considered positive controls for each assay performed.

### Fluorescent focus reduction virus neutralization (FFN) test

The VN Ab titers to Wa RVA in passive treatment pools, egg yolks, supplemented milks, and pig sera were determined by fluorescent focus neutralization (FFN) test as previously described [63]. For *in vitro* RVA fluorescent focus reduction assay using different RVA strains, a fourfold dilution of each VHH clone (3B2 or a non-related clone) was mixed with an equal volume of RVA containing 100 DICT. The VN titer was expressed as the reciprocal of the highest sample dilution that resulted in >80% reduction in the number of fluorescent foci. The 80% reduction criterion was selected in order to be more stringent than in protocols considering only 50% reduction.

### Isolation of mononuclear cells (MNCs) and Wa RVA-specific ELISPOT assay

Tissue samples of duodenum, jejunum, and ileum lamina propria were collected. The MLNs were collected and processed separately. The MNCs from blood and spleen were extracted to evaluate ASC responses in systemic lymphoid tissues. All the MNC suspensions were obtained as previously described for pig tissues and blood [82] and the purified cells from all tissues and blood were resuspended to a final concentration of 5×10^6^ MNC/ml in RPMI-1640 (GIBCO) supplemented with 10% FBS, 20 mM HEPES, 2 mM Glutamine, 1 mM sodium pyruvate, 0.1 mM non-essential amino acids, 100 IU/ml penicillin, 67 mg/ml streptomycin and 50 mg/ml gentamycin (E-RPMI). The cell viability of each MNC suspension was assessed by Trypan blue exclusion (in all cases it was >90%). An ELISPOT assay for quantification of Wa RVA specific IgM, IgA, IgG ASC was conducted to evaluate effector B-cell responses from all piglets at PID 21, as previously described [53], [63], [64], [82]. The spots were developed with a tetramethylbenzidine peroxidase substrate system (TMB, KPL, Kirkegaard & Perry Laboratories, Inc.).

### Statistical analysis

Fisher's exact test was used to compare proportions of animals with diarrhea and virus shedding among groups. The Kruskall-Wallis rank sum test (non-parametric) was used to compare days of onset and duration of diarrhea and virus shedding, cumulative diarrhea scores and cumulative titers of virus shed (area under the curve, AUC) among groups that were recorded from PID 0 to 21. Negative samples at a dilution of 1∶4 were assigned an arbitrary Ab titer of 2 for the calculation of geometric mean titers (GMTs). Neutralizing and isotype-specific Ab titers were log_10_-transformed prior to statistical analysis. Differences in Ab titers among groups were evaluated by comparison of means at four different time-points post virus inoculation (PID 0, 7, 14, 21). Multiple comparison test of repeated measures throughout time was done following Akaike criteria for the selection of covariance matrices [88], [89]. In further comparisons of treatments and post virus inoculation time-points, Šidák's correction was applied [90]. At PID 21, the ASC numbers were compared among groups using the Kruskall-Wallis rank sum test. Statistical significance was assessed at p<0.05 for all comparisons. Statistical analyses were conducted using Infostat statistical software and MedCalc version 11.1.1.0 statistical software. Statistical analysis is available upon request.
